# Examining the Link Between ADHD Symptoms and Menopausal Experiences

**DOI:** 10.1177/10870547251355006

**Published:** 2025-07-30

**Authors:** Lauren Chapman, Kanak Gupta, Myra S. Hunter, Eleanor J. Dommett

**Affiliations:** 1 King’s College London, UK

**Keywords:** inattention, hyperactivity/impulsivity, health-related quality of life, estrogen

## Abstract

**Objectives:**

Interest in the role of female hormones in ADHD has grown in recent years and, with an increasing number of women diagnosed with ADHD later in life, it is important hormonal changes across the lifespan are considered. This exploratory study examines the relationships between ADHD status (diagnosis and medication use) and symptoms, and menopause stage (pre/peri/post) and symptoms.

**Methods:**

Employing a cross-sectional approach, we recruited a sample of 656 women aged 45 to 60 years, of which 245 had an existing diagnosis of ADHD. Women completed several questionnaires assessing their ADHD symptoms (Adult Self-Report Scale, ASRS) and menopausal experiences (Women’s Health Questionnaire, WHQ; Menopause-Specific Quality of Life Questionnaire, MENQoL; Hot Flush Rating Scale, HFRS; Hot Flush Related Daily Interference Scale, HFDIS).

**Results:**

ANCOVA revealed no significant effects of an ADHD diagnosis or interaction effects between diagnosis and menopause stage after applying an FDR correction. Similarly, when medication was considered (i.e., non-ADHD, ADHD with medication, and ADHD without medication) there were no main effects of group or interaction effects with menopause stage.

**Conclusions:**

These results indicate women with ADHD do not experience greater menopausal complaints than women without at any menopausal stage. However, there were significant correlations between ADHD symptoms and menopausal complaints across all participants but at a group level, these were less prominent in those with ADHD, which could indicate different attribution of symptoms in women with ADHD. Future research should further explore menopause in women with ADHD considering longitudinal designs and qualitative studies to examine potential overlap of symptoms and symptom attribution.

## Introduction

ADHD, characterized by inattention, hyperactivity, and impulsivity impairing daily functioning, is the most common developmental condition (American Psychiatric Association, 2013). Although traditionally considered a condition outgrown after adolescence (Smith et al., 2024), it is now recognized to impact all ages with a global adult prevalence of around 3% (Song et al., 2021), making it critical that a lifespan approach is taken to ADHD. Despite this, a less conspicuous symptomology results in delayed diagnosis and treatment of adult ADHD, particularly in women (da Silva et al., 2020), and within the context of a wider crisis in adult ADHD care within the UK (Smith et al., 2024). Furthermore, women with ADHD are more prone to academic underachievement and lower self-esteem than their male counterparts (Young et al., 2020). Delays to diagnosis and treatment exacerbate adverse outcomes associated with ADHD, including increased unemployment, divorce rates, incarcerations, substance use disorders (SUDs), suicide attempts, and unintentional injuries, as well as poorer quality of life (Balazs & Kereszteny, 2017; Choi et al., 2022; Libutzki et al., 2023).

Several reasons have been suggested for the sex-discrepancies in diagnosis and treatment, including male-oriented bias within diagnostic criteria (Mowlem et al., 2019) and a lack of awareness by health professionals about ADHD characteristics in girls and women (Antoniou et al., 2021). Women with ADHD are also more likely to have co-occurring depression, anxiety, and eating disorders, which may mask ADHD symptoms and result in delays to diagnosis and appropriate treatment (Faheem et al., 2022; Groß-Lesch et al., 2016; Young et al., 2020). Irrespective of why women are disadvantaged, there are calls for research into ADHD to examine sex carefully and consider the role of reproductive hormones (Williamson & Johnston, 2015) with recognition that the interaction between sex hormones and neurotransmitters may be critical in ADHD (Pines, 2016).

Women undergo significant hormonal fluctuations over their lifespan. The menstrual cycle, a biological process for fertilization and pregnancy, involves cyclical fluctuations of ovarian hormones estrogen and progesterone (Williamson & Johnston, 2015). During the luteal phase before menstruation, estrogen levels decline to their lowest (Haimov-Kochman & Berger, 2014). The depletion of estrogen is associated with reduced dopamine, negative affect, and reduced cognitive functioning (Eng et al., 2024). Given this, it is perhaps unsurprising, that ADHD symptoms have been found to increase during periods of low estrogen within the menstrual cycle (Bürger et al., 2024), resulting in medication being less effective (Quinn, 2005) and calls to adjust ADHD medication in line with menstruation (de Jong et al., 2023; Roberts et al., 2018). However, by focusing on short-term menstrual cycles, research on ADHD to date has failed to capture the most long-lasting hormonal changes that occur during a woman’s lifespan, that is when she transitions out of her reproductive years. Indeed, although women undergo menstrual cycles for approximately 40 years, these typically follow predictable 28-day intervals to which the body can become accustomed (Harlow & Paramsothy, 2011). Even pregnancy, another significant hormonal change, is relatively brief lasting approximately 9 months, with hormonal stabilization typically within a year (Lopez-Gonzalez & Kopparapu, 2024). In contrast, the menopause transition can extend to 15 years (Peacock et al., 2024).

The menopause transition is a unique developmental stage due to the varied physical, endocrine, and psychological changes associated with it (Hilditch et al., 1996). It is divided into several stages: premenopause, perimenopause, and postmenopause. Premenopause refers to the reproductive years which are generally characterized by regular menstrual bleeding and stable estrogen. Perimenopause precedes menopause and is characterized by irregular menstruation and fluctuating hormones. During this period, estrogen levels fluctuate more dramatically than during menstrual cycles (Hoyt & Falconi, 2015). These fluctuations can cause vasomotor symptoms (e.g., hot flushes and night sweats), with over 80% of women experiencing these (Talaulikar, 2022) and it is these symptoms that are the primary reason women seek medical assistance (M. S. Hunter & Liao, 1995). While these typically persist for 1 to 6 years, they last up to 15 years in 10% to 15% of cases (Avis et al., 2015). Additional psychosocial symptoms, including heightened anxiety, disrupted sleep, brain fog, low libido, depressed mood, and poor memory, may resemble those associated with ADHD (Talaulikar, 2022) and can persist postmenopause. The severity and duration of menopausal symptoms vary greatly and are influenced by factors including ethnicity and intrapersonal and interpersonal aspects (Baber et al., 2016; O’Neill & Eden, 2012). The menopause refers to the last menstrual period, and 12 months after the final menstrual bleed, which occurs at an average of 51 years, and it is at this point women enter the postmenopause stage (Peacock et al., 2024).

For women with pre-existing ADHD, managing menopausal symptoms can be complex. Individuals with ADHD were initially believed to possess less dopamine and/or inefficiently use available dopamine (Oades, 2008). Whilst evidence for a hypo-dopaminergic state in ADHD is limited, there is certainly converging evidence for involvement of dopamine (MacDonald et al., 2024). Declining estrogen during the menopause transition may therefore interact with dopamine in a way that may intensify psychological symptoms such as emotional dysregulation, disorganization and inattention, impaired short-term memory, and increased risk of neuropsychiatric disorders (Dorani et al., 2021; Epperson et al., 2015). Accordingly, for women who had previously managed their ADHD symptoms well, possibly with medication, menopause may initiate a period of increasingly unregulated symptoms through altered estrogen-dopamine interactions. Despite recognition that hormonal changes may impact ADHD, a systematic review recently noted the very limited research investigating female reproductive hormones in ADHD and none of the studies focused on the menopause (Camara et al., 2022).

Although the review reported no studies examining the menopause in ADHD, interest in topic of how the menopause and ADHD co-occur or interact is increasing (Pines, 2016). For example, in a study examining women with and without autism spectrum disorder (ASD), ADHD traits were found to be positively correlated with menopausal complaints, suggesting that women, irrespective of a formal ADHD diagnosis, may display ADHD-like traits during midlife (Groenman et al., 2022). More recently, a qualitative study incorporating interviews with six women with combined diagnosis of ASD and ADHD indicated that struggling with the peri-menopause was often a catalyst for seeking an ADHD diagnosis (Craddock, 2024). Additionally, a study utilizing the Brown Attention Deficit Disorder Scale (BADDS) revealed that ADHD-like symptoms increased as women shift from pre to perimenopause, with a partial recovery postmenopause, but only if menopause was natural rather than surgical (Page et al., 2023). This study had a large sample size (*N* = 1,971) with a small proportion indicating that they had a prior diagnosis of ADHD (*N* = 203, 10.2%). However, this group was not analyzed separately but rather diagnosis controlled for in regression models and no measure of menopause symptoms was made, collecting only menopause stage, meaning it is not possible to compare menopausal experience in women with and without ADHD from this work.

Studies have investigated the effects of non-stimulant and stimulant medication normally prescribed for ADHD on midlife cognitive function. Six weeks of treatment with atomoxetine significantly reduced working memory and attention deficit scores on the Brown Attention Deficit Disorder Scale (BADDS) compared to baseline, alongside improving concentration in 16 peri and postmenopausal women without ADHD (Epperson et al., 2011). Similarly, lisdexamfetamine significantly improved memory, multitasking, and problem-solving abilities in 32 peri and postmenopausal women experiencing midlife cognitive function impairments in the absence of existing ADHD (Epperson et al., 2015). Collectively, these few studies support a relationship between ADHD and menopause, and the potential of ADHD treatments to help ease cognitive symptoms in midlife. They also, however, highlight a lack of research investigating menopausal symptoms in those with ADHD.

The lack of research examining the menopause in ADHD, in spite of the longevity of the menopause and periods of significant disruption, is likely to be related to the fact that, as a topic, the menopause is under-researched, under-treated, and stigmatized (Dintakurti et al., 2022). For example, a UK survey of 947 perimenopausal women revealed that over 60% did not feel informed at all about the menopause and many reported feeling unsupported if/when they did seek medical help (Harper et al., 2022). The inadequate management of the menopause may in part stem from deficient menopause education in UK medical school curricula (Harper et al., 2022), leaving clinicians ill-equipped to address women’s concerns (Tariq et al., 2023). This is problematic, as negative attitudes toward the menopause often aggravate symptoms. For instance, the European Menopause Survey revealed that UK women report more severe symptoms and a lower quality of life than Spanish and French women (Genazzani et al., 2006), noting lower symptom prevalence in cultures viewing menopause as a natural ageing process rather than a taboo (Tariq et al., 2023). This suggests that much like ADHD services being in crisis in the UK, menopause care is also lacking. Collectively, this could create a significant unmet health need in midlife women with ADHD.

The first step in addressing this health need is to better understand the experiences of the menopause for women with ADHD. This understanding would allow more targeted support for this cohort if needed. As such, the current exploratory study aims to compare menopausal experiences between women with an existing diagnosis of ADHD and those without. Additionally, given the possibility that ADHD medication may impact both ADHD and menopausal complaints we will also examine the effect of ADHD medication on menopause by comparing women with ADHD on and off medication with those without ADHD. Specifically, we firstly hypothesized that women with ADHD, will experience significantly worse menopausal complaints than women without ADHD, moderated by menopausal stage (pre, peri, and post; H1). Related to this was our second hypothesis which allowed us to drill into the effects of medication by hypothesizing that women with ADHD, both on and off medication, will experience significantly worse menopausal complaints than women without ADHD, moderated by menopausal stage (pre, peri, and post; H2). Finally, we suggest that ADHD symptoms will correlate with menopausal complaints in all women, but the strength of correlation may vary between groups according to diagnostic status and use of medication (H3).

## Methods

### Study Design and Participants

This was a cross-sectional investigation carried out between October 2023 and June 2024. All data were collected via the online survey platform Qualtrics which allows for anonymous data collection. Using this platform, we were able to guard against fraudulent entries that can impact online research by preventing indexing in search engines, using automated bot detection and ReCAPTCHA scores, combined with removal of any responses completed in less than one third of the median response time (van Tilburg et al., 2022). The research was approved in advance by King’s College London Ethical Review Committee (LRS/DP-23/24-39789). All participants provided informed consent to participate and for the research to be published.

Participants were recruited via convenience sampling using online advertisements across several digital platforms including the university’s volunteer recruitment website and mailing list, the laboratory website and mailing list, the volunteer platform “Call for Participants,” and several social media channels (X, Instagram, and Facebook). Advertisements were also shared through ADHD UK, and Menopause Matters. Each advertisement contained a brief study overview and a direct link to a participant information sheet and consent form. Participants provided electronic consent and verified their eligibility by answering questions regarding inclusion and exclusion criteria prior to accessing the survey. While no individual compensation was provided, participants were offered entry into a prize draw for shopping vouchers valued at £100, £50, or £25 upon survey completion. Those wishing to do this were redirected to a separate survey to ensure submitted email addresses could not be paired with the survey responses.

Participants were eligible if they were: (i) biologically female and therefore able to experience menopause, (ii) aged between 45 and 60 years to encompass the period during which most women experience the menopause transition (Talaulikar, 2022), and (iii) residing in the UK. We limited our sample to the UK given the significant variation in healthcare practices and ADHD diagnostic criteria between countries (Namazi et al., 2019). Women were excluded if they had undergone surgical menopause (oophorectomy), because the abrupt cessation of hormones often causes more intense vasomotor symptoms and mood disorders (Kingsberg et al., 2023) and has been shown to result in distinct experiences including those related to cognitive functioning (Page et al., 2023). Additionally, women currently using menopausal hormone therapy (MHT), or contraceptive pills were excluded. Hormone therapy can alleviate symptoms (Al-Safi & Santoro, 2014), and thus potentially confound the assessment. Continuous contraceptive use similarly disrupts natural fluctuations (Guerin et al., 2022). Hence, these exclusions ensured findings that reflect the natural menopausal process.

Since this is the first study to directly compare menopausal symptoms in women with and without ADHD, no prior data was available to guide sample size calculations. However, given the research suggesting an overlap between ADHD and menopausal symptoms (Epperson et al., 2015), a small to medium effect size was assumed. Sample size was determined using G*Power. For H1 (ANCOVA), assuming an effect size of *f* = 0.20, power (β) = .95, and a critical *p*-value of .05, a sample of 390 participants was required. For H2 (ANCOVA), assuming an effect size of *f* = 0.20, power (β) = .95, and a critical *p*-value of 0.05, a sample of 470 participants was required. For H3, with an effect size of *r* = 0.30, power (β) = .95, and a critical *p*-value of .05, a sample of 138 per correlation was required.

## Measures

### Demographic and General Health Assessment

On accessing the survey, participants first provided demographic information, specifically, age, ethnicity, relationship status (single, partner, married/co-habiting, divorced/separated, or widowed), educational achievement (left school at age 16 or 18 years or degree/professional qualifications), and current employment (working full-time, part-time, or not working).

To assess general health, participants reported their number of visits to a general practitioner (GP) and hospital appointments within the previous 6 months. They also indicated whether they had sought help specifically for menopause-related symptoms during this time (yes/no). Menopause stage was determined based on menstruation pattern: regular periods in the past 6 months (premenopausal), irregular periods in the past 6 months (perimenopausal), or no periods for at least 12 months (postmenopausal). They then reported any history of MHT (yes/no). Participants were also asked to list any medical diagnoses or health problems that they had, other than ADHD in a free-text entry box.

### ADHD Diagnosis and Symptoms

Respondents reported whether they had a formal ADHD diagnosis made by a healthcare professional (yes/no). Those diagnosed indicated whether they were currently prescribed ADHD medication (yes/no) and specified the type (stimulant/non-stimulant/other) as well as adherence measured using a previously developed two-item measure (Safren et al., 2007). Finally, participants indicated whether they received any other form of ADHD treatment from healthcare professionals (yes/no). Irrespective of whether individuals reported a diagnosis of ADHD, all completed the Adult ADHD Self-Report Scale (ASRS; Kessler et al., 2005), which is a validated self-report scale for ADHD-like behaviors. The survey contains 18 statements where participants rate the frequency of symptoms (0 = “Never” to 4 = “Very Often”) for questions such as: “How often do you misplace or have difficulty finding things at home or at work?”. The scale divides into two subscales, each containing 9-items: Hyperactivity-Impulsivity (α = .901) and Inattentive (α = .863). Responses to all 18 items are summed to produce a total score, with higher scores reflecting a stronger presence of ADHD traits. The ASRS demonstrated excellent internal consistency (α = .926).

### Menopausal Experience

Given the paucity of research in this area, several surveys were used to assess menopausal experience. Firstly, the Women’s Health Questionnaire (WHQ) was used to assess health-related quality of life (QoL) across six core domains: anxiety/depressed mood (seven items, α = .844), wellbeing (four items, α = .67), sleep (two items, α = .392), vasomotor symptoms (two items, α = .799), somatic symptoms (five items, α = .757), and memory/concentration (three items, α = .666; M. Hunter, 1992). The revised 23-item version of the WHQ was used, which shows superior cross-sectional psychometric properties compared to the original 36-item version (Girod et al., 2006). The impact of each item on their QoL over the past few days was rated on a 4-point scale (1 = “Yes, definitely” to 4 = “No, not at all”). Two optional domains from the original WHQ on sexual and menstrual symptoms were also included which used the same 4-point scale. Those in a sexual relationship (yes/no) rated their sexual activity (three items, α = .591) and those menstruating in the past 6 months answered the menstruation domain (four items, α = .724). Each subscale was averaged to produce a single score. Higher scores indicated a better QoL.

Secondly, the Menopause-Specific Quality of Life Questionnaire (MENQoL) was used to assess the impact of menopausal symptoms on QoL (Hilditch et al., 1996). The questionnaire comprises 29 items in four domains: vasomotor (3 items, α = .789), psychosocial (7 items, α = .828), physical (16 items, α = .838), and sexual (3 items, α = .695). The vasomotor domain assessed hot flushes, night sweats, and sweating. The psychosocial domain evaluated psychological wellbeing, including anxiousness and nervousness. The physical domain included bloating and tiredness. The sexual domain assessed sexual desire, vaginal dryness, and intimacy avoidance. Participants indicated whether they experienced each symptom in the past month (yes/no) and the level of bother on a 7-point Likert scale (0 = “Not at all” to 6 = “Extremely bothered”). Items are converted onto a scale from 1 to 8 where 1 = no symptom experienced and 8 = experienced and rated as 6 on the bothered scale, that is, the yes/no part of the item is combined with the bothersome part. Higher scores indicated a worse QoL.

Thirdly, two scales related to hot flushes were used. The Hot Flush Rating Scale (HFRS), which is a well-validated self-report scale assessing the frequency, severity, and problem-rating of hot flushes and night sweats, was used (M. S. Hunter & Liao, 1995). Participants reported via a free-text option how often they experienced hot flushes and were awakened by night sweats in the past week. Hot flush severity was then rated on a 3-point scale (1 = “Mild” to 3 = “Severe”). The problem-rating was then calculated by averaging three items assessing how much individuals consider their flushes/sweats to be a problem, how much distress they cause and how much they interfere with daily activities, all rated on a 10-point scale (1 = “Not distressed/not at all” to 10 = “Very distressed/very much affected,” α = .899). Higher scores on aspects of the HFRS indicated more frequent, severe, and problematic vasomotor symptoms. Additionally, the Hot Flush Related Daily Interference Scale (HFDIS) was used to assess the interference with daily life, including social activities and work on a 11-point scale (0 = “Do not interfere” to 10 = “Completely interfere”; Carpenter, 2001). All 10 items were averaged, and a higher score indicated greater interference with daily life on the HFDIS. The HFDIS demonstrated excellent internal consistency (α = .961).

### Statistical Analysis

All data was extracted from Qualtrics and imported to IBM SPSS Statistics version 29.0 for analysis. Items 7, 10, 18, 21, and the second item in the WHQ sexual domain were reverse scored. The frequency of GP and hospital visits was recoded according to predefined rules to standardize responses to these free-text questions. When a range was provided (e.g., 10–20) the middle value was taken (i.e., 15). If only an upper or lower limit was given (e.g., at least 10) the stated value was used (i.e., 10). Additional health conditions were grouped into overarching categories (see Supplemental Material 1). To prepare the data for analysis, individual item scores were averaged into a composite score for each subscale. Missing data was handled by including only participants with complete data for each menopausal subscale in the respective analyses, maximizing the use of all available data. Variations in the total number of participants analyzed are detailed in the results. Statistical significance was set at a threshold of *p* < .05 (5%). Reliability of the measures was assessed using Cronbach’s alpha (α), with a value of .6 or higher considered reliable.

To characterize the sample, descriptive statistics, including frequencies (*n*, %), means (*M*), and standard deviations (*SD*), were calculated to summaries demographic and clinical variables across groups (non-ADHD, medicated ADHD, and unmedicated ADHD). Normality was assessed using the Shapiro-Wilk test, alongside visual inspection of distribution histograms and measures of skewness and kurtosis. The distributions were deemed suitable for parametric testing. Chi-square tests assessed any differences or association in categorical demographic variables (e.g., menopausal stage) across groups. A one-way ANOVA assessed differences in the continuous variables (e.g., age, ASRS score) between groups with post-hoc Bonferroni tests.

To test H1, a 2 × 3 factorial Analysis of Covariance (ANCOVA) assessed the main effects and interaction effects of group (ADHD vs. non-ADHD) and menopausal stage (pre/peri/postmenopausal) on each menopausal complaint subscale, while controlling for demographic variables with significant differences between groups (age, education, GP visits, hospital visits, and presence of additional diagnoses). To test H2, the same analysis was used but considering three groups (non-ADHD, medicated ADHD, and unmedicated ADHD), that is, a 3 × 3 factorial Analysis of Covariance (ANCOVA) assessed the main effects and interaction effects of group and menopausal stage (pre/peri/postmenopausal) on each menopausal complaint subscale, while controlling for demographic variables with significant differences between groups (age, education, GP visits, hospital visits, and presence of additional diagnoses). To test H3, bivariate Pearson’s *r* correlations first assessed the relationships between ADHD symptoms as measured by the total ASRS score and menopausal complaint scales in the three for the whole population and then for the three different groups. The total ASRS score was used rather than the subscales due to the subscales being strongly correlated (*r* > .6). To compare the size of the correlations between the groups, a Fisher’s *r* to *z* transformation was carried out. This common approach for comparing correlations between independent groups enables the z scores created from the transformed correlation coefficients to be analyzed for statistical significance with the Fisher’s *z* test (Lenhard & Lenhard, 2014) and has been previously used in ADHD research (Kebir et al., 2009).

Given the exploratory nature of this study and the conduction of planned comparisons, we did not apply a standard correction for multiple comparisons, for example, Bonferroni, in our hypothesis testing because we recognize that this can obscure meaningful associations in real-world data by overemphasizing the role of chance as the first-order explanation for observed phenomena (Rothman, 1990). However, to strike a balance between exploratory work and robust findings, we calculated the Benjamini-Hochberg False Discovery Rate (FDR) correction to account for the increased risk of false positives that can arise from conducting multiple statistical tests (Benjamini & Hochberg, 1995). This correction helps balance the need to control for false discoveries while minimizing the loss of potentially meaningful associations (Benjamini, 2010). Instead of calculating an adjusted *p*-value for the individual tests, the FDR correction calculates adjusted critical *p*-values. For example, if six tests were completed the resultant *p*-values are ranked in descending order and compared to six different critical p-values. The first critical value (α_1_) is .05, with the next critical value (α_2_) being α_1_ – (α_1_/6) and the third (α_3_) being α_2_ – (α_1_/6). Consequently, *p*-values are reported as calculated and shown in bold if they are significant against the FDR corrected α.

## Results

### Sample Characteristics

Of an initial 1,076 participants, 418 (38.84%) were excluded for not finishing the survey, in line with typical attrition rates for online surveys of this length. An additional two were excluded for incomplete ASRS data. This resulted in a final sample of *N* = 656 participants, including 245 declaring an existing diagnosis of ADHD (107 medicated ADHD-M and 138 unmedicated ADHD-U), and 411 declaring that they did not have an ADHD diagnosis (non-ADHD). As expected, there were significant group differences for the Total ASRS, *F*(2, 653) = 43.815, *p* < .001, η^2^ = .118, with both ADHD-M (*M* = 36.24, *SD* = 9.31) and ADHD-U (*M* = 37.38, *SD* = 8.34) scoring significantly higher than non-ADHD (*M* = 28.47, *SD* = 12.37, *p* < .001). There were no significant differences between the two ADHD groups (*p* = 1.00). Similarly, there were significant differences in the ASRS inattention subscale, *F*(2, 653) = 37.814, *p* < .001, η^2^ = .104, with both ADHD-M (*M* = 20.34, *SD* = 4.79) and ADHD-U (*M* = 20.42, *SD* = 4.63) scoring significantly higher than non-ADHD (*M* = 15.98, *SD* = 7.04, *p* < .001). There were no significant differences between the ADHD groups (*p* = 1.00). Finally, there were also significant differences for the hyperactive-impulsive subscale, *F*(2, 653) = 36.216, *p* < .001, η^2^ = .118, with ADHD-M (*M* = 15.91, *SD* = 5.25) and ADHD-U (*M* = 16.96, *SD* = 5.00, *p* < .001) scoring significantly higher than non-ADHD (*M* = 12.49, *SD* = 6.32). There were no significant differences between the ADHD groups (*p* = .504). Average medication adherence for ADHD-M was 79% (*SD* = 29%), with 97 (90.7%) taking stimulants, 6 (5.6%) taking non-stimulants, and 4 (3.7%) using other treatments, including selective serotonin reuptake inhibitors.

A summary of the demographic characteristics of all groups is provided in Table 1. There were no significant differences in ethnicity, relationship status, or employment. However, there were differences in education level, with those with ADHD more likely to have left school at a younger age. Additionally, individuals with ADHD were more likely to have a higher number of GP and hospital visits. Whilst this could be attributed to their ADHD, it may also relate to other health conditions, which were more common in those with ADHD. A breakdown of the health complaints reported is given in Supplemental Table 1. The huge variety in conditions and variation in detail provided means no further analysis was completed on these conditions, and this was instead considered as a categorical variable (presence of additional health conditions vs. absence). Finally, there were some differences in age with post-hoc comparisons using the Bonferroni test revealing that non-ADHD were significantly older than ADHD-M (*p* < .001) but not ADHD-U (*p* = .388) and there was no difference between the two ADHD groups (*p* = .060). Note that the same variables differed significantly between groups when non-ADHD were compared to all those with ADHD (i.e., a combined sample of ADHD-M and ADHD-U).

**Table 1.** Baseline Demographic Characteristics of Participants by ADHD Status.

Factor	Non-ADHD		ADHD-M		ADHD-U		*χ* ^2^	*p*
	*n* = 411		*n* = 107		*n* = 138			
	*n*	%	*n*	%	*n*	%		
Ethnicity^a^								
White	377	91.7	101	94.4	123	89.1	2.19	.334
Non-white	34	8.3	6	5.6	15	10.9		
Relationship status								
Single	77	18.7	18	16.8	22	15.9	1.87	.931
Married/partner	283	68.9	77	72.0	100	72.5		
Divorced/separated	43	10.5	10	9.3	15	10.9		
Other	8	1.9	2	1.9	1	0.7		
Education								
Left school at 16	18	4.4	11	10.3	17	12.3	14.61	.024
Left school at 18	45	10.9	11	10.3	8	5.8		
Degree	319	77.6	79	73.8	103	74.6		
Other	29	7.1	6	5.6	10	7.2		
Employment								
Full-time	200	48.8	43	40.2	56	40.6	6.20	.185
Part-time	131	32.0	34	31.8	49	35.5		
Not working	79	19.3	30	28.0	33	23.9		
GP visits								
0–2	147	35.8	18	16.8	34	24.6	26.93	<.001
3–5	118	28.7	27	25.2	35	25.4		
6–8	68	16.5	24	22.4	27	19.6		
9+	78	19.0	38	35.5	42	30.4		
Hospital visits								
0–2	3	0.7	0	0	0	0	22.06	.001
3–5	344	83.7	71	66.4	106	76.8		
6–8	37	9.0	16	15.0	15	10.9		
9+	27	6.6	20	18.7	17	12.3		
Other medical conditions	259	63.0	86	80.4	114	82.6	25.46	<.001
	*M*	*SD*	*M*	*SD*	*M*	*SD*	*F*	*p*
Age (years)	51.09	4.21	49.21	3.79	50.46	4.17	8.76	<.001^b^

a Ethnicity is categorized as White/Non-white to meet chi-square test assumptions. More detailed ethnic categories led to over 20% of cells with expected counts of less than 5.

b ANOVA η^2^ = .26.

Table 2 presents menopause-related categorical variables by group. Chi-square analyses indicated no significant differences in menopause stage between groups. However, there was a trend-level (*p* = .05) difference for whether they had previously sought help for the menopause with ADHD-M more likely to have done so. There was also a significant difference in previous MHT use, such that women with ADHD (ADHD-M and ADHD-U) were more likely to have received this.

**Table 2.** Menopause Status Details for All Participants.

Factor	Non-ADHD		ADHD-M		ADHD-U		*χ* ^2^	*p*
	*n* = 411		*n* = 107		*n* = 138			
	*n*	%	*n*	%	*n*	%		
Menopause stage								
Premenopause	83	20.2	27	25.2	23	16.7	3.12	.537
Perimenopause	162	39.4	39	36.4	53	38.4		
Post menopause	166	40.4	41	38.3	62	44.9		
Help sought								
Yes	130	31.6	47	43.9	44	31.9	6.00	.050
No	281	68.4	60	56.1	94	68.1		
History of MHT								
Yes	60	14.6	27	25.2	31	26.3	8.88	.012
No	351	85.4	80	74.8	107	77.5		
Experiencing vasomotor symptoms^a^								
Yes	243	59.3	63	58.9	79	57.2	0.18	.916
No	167	40.7	44	41.1	59	42.8		

a One person without ADHD omitted to answer this question.

### The Effect of ADHD Diagnosis on Menopause

The 2 × 3 ANCOVA analysis revealed no significant main effects of a reported diagnosis of ADHD on menopause measures after FDR correction was applied (Table 3). Perhaps unsurprisingly, the ANCOVA did reveal significant effects of menopausal stage on several complaints across the different instruments. Given this is not central to the hypothesis, these results can be found in Supplemental Table S2 and are only described briefly here. On the WHQ, results indicated that vasomotor related quality of life significantly declined with each consecutive menopausal stage. Premenopausal women also reported significantly higher memory/concentration related quality of life compared to peri but not postmenopausal women, suggesting some recovery of this component in the final stage. MENQoL data revealed similar findings for vasomotor symptoms with premenopausal women experiencing less difficulties than perimenopause and postmenopause. Additionally, on the MENQoL, perimenopausal women had significantly more psychosocial symptoms than both premenopausal and postmenopausal women. Data from the HFRS corroborated the WHQ and MENQoL showing premenopausal women had fewer hot flushes and night sweats than postmenopausal women and this was also reflected in the problem rating and the HFDIS scores. Additionally, and critical for addressing H1, there were no significant interactions between reported diagnosis and menopausal stage for any measure after FDR was applied (see Supplemental Table S3). These results suggest that an ADHD diagnosis does not impact menopausal complaints.

**Table 3.** Main Effects of ADHD Status on Menopausal Complaints With Significant Effects Indicated in Bold, Although It Should Be Noted That These Did Not Remain Significant After FDR Correction.

Menopausal complaints	Subscales	Non-ADHD *M* (*SD*)	ADHD *M* (*SD*)	*df*	*F*	*p*	η_p_^2^
WHQ	Anxiety/depressed	2.42 (0.68)	2.20 (0.63)	1, 632	2.263	.133	.004
	Wellbeing	2.71 (0.56)	2.55 (0.60)	1, 633	.586	.444	.001
	Sleep	2.13 (0.81)	1.95 (0.75)	1, 634	5.007	.026	.008
	Vasomotor	2.48 (0.99)	2.46 (0.98)	1, 636	1.032	.310	.002
	Somatic	2.28 (0.68)	2.12 (0.64)	1, 633	.000	.993	.000
	Memory/concentration	1.86 (0.66)	1.62 (0.49)	1, 633	7.855	.005	.012
	Sex	2.41 (0.73)	2.32 (0.80)	1, 357	.986	.322	.003
	Menstrual	1.97 (0.73)	1.92 (0.69)	1, 372	.100	.752	.000
MENQoL	Vasomotor	3.46 (2.14)	3.67 (2.22)	1, 639	.081	.776	.000
	Psychosocial	5.10 (1.71)	5.57 (1.56)	1, 639	1.373	.242	.002
	Physical	4.59 (1.39)	4.84 (1.39)	1, 639	.731	.393	.001
	Sexual	3.68 (2.18)	3.59 (2.16)	1, 639	.736	.391	.001
HFRS	HF frequency/week	15.63 (21.44)	11.62 (17.21)	1, 309	1.904	.169	.006
	NS frequency/week	9.89 (10.31)	8.71 (10.59)	1, 315	.965	.327	.003
	HF Severity	1.06 (1.03)	.95 (.82)	1, 371	.858	.355	.002
	NS Severity	1.03 (0.88)	1.09 (.89)	1, 363	.361	.548	.001
	Problem Rating	3.66 (2.23)	4.21 (2.50)	1, 370	1.527	.217	.004
HFDIS	Total	3.25 (2.64)	3.80 (2.98)	1, 350	.751	.387	.002

### The Effect of ADHD Diagnosis and Medication Status on Menopause

The analysis distinguishing between women reporting use of medication and those who were medication free, along with those without ADHD, that is, the 3 × 3 ANCOVA, revealed no significant main effects of ADHD group on menopause measures after FDR correction was applied (Table 4). These results imply that even when medication status is considered there is no significant difference in menopausal complaints after FDR correction between women with and without ADHD, on and off medication when all menopausal stages are combined. Given that this ANCOVA partially replicated the analysis for H1, the significant main effects of menopause stage remained the same as reported above (Supplemental Table S2). Critically for H2, the ANCOVA did not reveal any significant interactions that withstood the FDR corrections (Supplemental Table S4).

**Table 4.** Main Effects of ADHD Status Using Three Groups on Menopausal Complaints.

Menopausal complaints	Subscales	Non-ADHD *M* (*SD*)	ADHD-M *M* (*SD*)	ADHD-U *M* (*SD*)	*df*	*F*	*p*	η_p_^2^
WHQ	Anxiety/depressed	2.42 (0.68)	2.21 (0.62)	2.19 (0.64)	2, 629	2.252	.106	.007
	Wellbeing	2.71 (0.56)	2.53 (0.58)	2.57 (0.61)	2, 630	.530	.589	.002
	Sleep	2.13 (0.81)	2.05 (0.74)	1.87 (0.75)	2, 631	3.544	.029	.011
	Vasomotor	2.48 (0.99)	2.45 (0.95)	2.46 (1.00)	2, 633	.443	.642	.001
	Somatic	2.28 (0.68)	2.13 (0.65)	2.11 (0.64)	2, 630	.202	.817	.001
	Memory/concentration	1.86 (0.66)	1.64 (0.48)	1.61 (0.51)	2, 630	3.385	.035	.011
	Sex	2.41 (0.73)	2.44 (0.81)	2.25 (0.80)	2, 354	1.057	.349	.006
	Menstrual	1.97 (0.73)	1.94 (0.65)	1.91 (0.72)	2, 370	.378	.686	.002
MENQoL	Vasomotor	3.46 (2.14)	3.71 (2.17)	3.62 (2.56)	2, 638	.045	.956	.000
	Psychosocial	5.10 (1.71)	5.55 (1.61)	5.59 (1.67)	2, 638	2.105	.123	.007
	Physical	4.59 (1.39)	4.63 (1.44)	5.01 (1.33)	2, 638	3.343	.036	.010
	Sexual	3.68 (2.18)	3.38 (2.07)	3.75 (2.22)	2, 638	1.941	.144	.006
HFRS	HF frequency/week	15.63 (21.44)	10.12 (13.31)	12.90 (19.99)	2, 306	1.464	.233	.009
	NS frequency/week	9.89 (10.31)	6.79 (7.21)	10.35 (12.62)	2, 312	2.179	.115	.014
	HF severity	1.06 (1.03)	0.86 (0.95)	1.03 (0.98)	2, 368	1.736	.178	.009
	NS severity	1.03 (0.88)	1.18 (0.97)	1.01 (0.81)	2, 368	.305	.737	.002
	Problem rating	3.66 (2.23)	3.95 (2.57)	4.44 (2.43)	2, 367	2.058	.129	.011
HFDIS	Total	3.25 (2.64)	3.54 (2.92)	4.01 (2.79)	2, 347	1.425	.242	.008

*Note*. It should be noted that no effects remained significant after application of the FDR correction.

### The relationship between ADHD traits and menopausal complaints

The correlations between total ASRS scores and menopausal complaint subscales were examined using Pearson’s *r* and are displayed in Table 5. When considering all participants, all WHQ measures, except vasomotor-related quality of life revealed significant negative correlations, that is, higher ADHD symptoms were associated with poorer quality of life. MENQoL results aligned with this for all measures, including vasomotor, indicating that across all women, as ADHD traits increased so did menopausal difficulties. For the HFRS and HFDIS, there was a significant positive correlation between problem rating and total interference score. These results remained significantly even after FDR correction.

**Table 5.** Correlations Between ADHD Symptoms as Measured by the ASRS and Menopausal Complaints in All Women Across All Menopausal Stages and Then for the Three Groups.

Menopausal complaints	Subscales	All participants			Non−ADHD			ADHD−M			ADHD−U		
		*n*	*r*	*p*	*n*	*r*	*p*	*n*	*r*	*p*	*n*	*r*	*p*
WHQ	Anxiety/depressed	656	−.427	**<.001**	410	−.450	**<.001**	104	−.213	.030	135	−.343	**<.001**
	Wellbeing	649	−.340	**<.001**	409	−.357	**<.001**	105	−.334	**<.001**	136	−.190	.027
	Sleep	650	−.221	**<.001**	409	−.197	**<.001**	104	−.214	.029	138	−.165	.053
	Vasomotor	651	−.071	.071	147	−.072	.147	106	−.093	.345	137	.061	.479
	Somatic	653	−.310	**<.001**	407	−.345	**<.001**	106	−.144	.140	137	−.181	.034
	Memory/concentration	650	−.545	**<.001**	408	−.568	**<.001**	106	−.355	**<.001**	136	−.379	**<.001**
	Sex	369	−.124	.**017**	229	.121	.067	56	−.067	.624	84	−.105	.344
	Menstrual	384	−.169	**<.001**	244	−.181	**<.001**	64	−.179	.157	76	−.107	.359
MENQoL	Vasomotor	656	.146	**<.001**	411	.160	**<.001**	107	−.139	.153	138	.074	.385
	Psychosocial	656	.447	**<.001**	411	.498	**<.001**	107	.297	.**002**	138	.222	.009
	Physical	656	.284	**<.001**	411	.319	**<.001**	107	.211	.029	138	.119	.165
	Sexual	656	.099	.**012**	411	.133	.**007**	107	.072	.461	138	.058	.500
HFRS	HF frequency/week	321	.083	.138	198	.218	.**002**	57	.086	.523	66	−.255	.039
	NS frequency/week	328	.034	.535	210	.112	.107	54	.087	.534	64	−.150	.238
	HF severity	384	.052	.308	242	.110	.087	63	.121	.345	79	−.102	.371
	NS severity	376	.007	.898	238	−.029	.660	62	.027	.833	76	.081	.488
	Problem rating	383	.230	**<.001**	242	.224	**<.001**	63	.195	.126	78	.154	.178
HFDIS	Total	363	.273	**<.001**	231	.311	**<.001**	58	.344	.**008**	74	.031	.796

*Note.* Bolded *p*-values indicate that this correlation remained significant after FDR correction. n = number of participants with complete data for each subscale. *r* *=* Pearson correlation coefficient.

Examination of the correlations within the different groups revealed slightly different patterns. For those without ADHD, the correlations, largely mimicked the overall group, likely because this group made up the largest single group within the population. Notable differences between the whole population and the non-ADHD group were a lack of correlation between the WHQ sex measure and ASRS score for the non-ADHD group and the presence of a positive correlation between ASRS score and hot flush frequency. Far fewer significant correlations were found for the two ADHD groups. For those on medication, only the wellbeing and memory/concentration subscales of the WHQ showed significant negative correlations with the ASRS, whilst on the MENQoL, only the psychosocial subscale significantly correlated with ASRS scores. There was a significant positive correlation with HFDIS and ASRS in this group, mimicking the overall sample findings. For those not receiving medication two subscales of the WHQ (anxiety/depression and memory/concentration) showed significant correlations. Supplemental Table S5 shows the *p*-values from Fisher’s *z* tests to determine whether the correlations differed between the groups. After FDR correction, only four significant differences remained between the groups. The correlations between the ASRS and anxiety and depression and memory/concentration on the WHQ were significantly smaller in the ADHD-M group compared to the non-ADHD group. Additionally, the un-medicated ADHD group also showed significantly smaller correlations to the non-ADHD group for memory/concentration (WHQ) and the psychosocial subscale of the MENQoL.

## Discussion

The aim of this study was to examine whether women with ADHD experience worse menopausal complaints than those without (H1), whether the use of medication impacts this (H2), and whether ADHD symptoms correlate with menopausal complaints (H3).

In assessing H1, we described main effects of ADHD group (Table 3) and menopausal stage (Supplemental Table S2) as well as interaction effects (Supplemental Table S3), with the main effects of ADHD group and interaction effects key to testing the hypothesis. For both, there were no significant effects after FDR correction. These findings imply that a diagnosis of ADHD is not associated with a worse menopausal experience contrary to our hypothesis. Arguably these findings are unexpected because ADHD is associated with several difficulties that overlap with menopausal complaints. For example sleep problems are commonly reported in ADHD (Kooij et al., 2001; van der Ham et al., 2024), although these may be associated with stimulant use rather than ADHD per se (Schneider & Enenbach, 2014). Similarly, memory and concentration difficulties are found in ADHD (Alderson et al., 2013; Skodzik et al., 2017) and various physical difficulties such as gastrointestinal problems (Leader et al., 2022), muscle or joint pains (Stray et al., 2013) are common, all of which could result in heightened experiences of similar symptoms in menopause. The lack of effects found for H1 could have resulted from the combining of those on and off medication into a single ADHD group, potentially because the medication offered some protection against both ADHD and menopause symptoms (Epperson et al., 2011; Epperson et al., 2015). To examine this, and test H2, a three-group comparison was conducted. This also revealed no significant main effects of ADHD status or interaction effects, indicating that the lack of effects in H1 was not due to the impact of medication use.

The analysis for H1 and H2 revealed significant main effects of menopause stage for various measures as would be expected. Vasomotor symptoms as measured by the WHQ and the MENQoL were found to be worse during the peri and postmenopausal stages compared to premenopause. These findings withstood FDR. The HFRS partially aligned with this showing that premenopausal women had fewer hot flushes and night sweats compared to perimenopausal women but not postmenopausal. The problem rating also showed a similar pattern. Interestingly, the HFDIS indicated that there was no significant difference between pre and perimenopause for daily interference by vasomotor symptoms but there was between pre and postmenopause. Overall, these findings align with what we would expect, that is, that vasomotor symptoms are a common feature of the perimenopause stage (Hoyt & Falconi, 2015) that can persist postmenopause (Avis et al., 2015). Memory and concentration as measured by the WHQ declined from pre to perimenopause and recovered during the postmenopausal stage, aligning with a recent review examining cognition during the perimenopause which indicates that memory and attention decline at this time (Metcalf et al., 2023). Psychosocial complaints as measured by the MENQoL, which includes mood, cognition and relationships with others, showed a similar pattern with difficulties encountered during the perimenopause.

To test H3, we calculated Pearson’s correlation coefficient for the relationship between the total ASRS and the various menopause scales for all groups combined and the three distinct groups. When considering the whole sample, various significant correlations were found across all menopause measures and the ASRS, but when considering the groups separately and only correlations that remained significant after FDR correction, more significant relationships were found between menopause measures and the ASRS in women without ADHD. In this group, correlations indicated that worse ADHD symptom scores were associated with worse menopausal complaints across all aspects of menopause except sex, on at least one scale. This aligns with prior work which examined the relationship between ADHD traits and menopause complaints in women with ASD (Epperson et al., 2011, 2015; Groenman et al., 2022). For women with ADHD on medication, correlations revealed that worse ADHD symptoms were associated with poorer wellbeing, memory and concentration, psychosocial symptoms, and hot flush interference. For those not on medication, ADHD symptoms were associated with worse anxiety and depression and memory and concentration.

Given that ADHD is often associated with anxiety and depression, particularly in women (Fuller-Thomson et al., 2016; Wicherkiewicz & Gambin, 2024), these correlations with anxiety/depression, wellbeing, and psychosocial aspects of the menopause across the ADHD groups are not surprising. The weaker correlations for anxiety/depression and memory/concentration in the ADHD-M group compared to the non-ADHD group could reflect the benefits of ADHD medication on these components. However, this is unlikely to fully explain the reduced ASRS-memory/concentration correlation because a similar reduction is found in those unmedicated. One explanation could be how the symptoms are attributed. Women with ADHD may attribute difficulties in memory and concentration to their ADHD rather than as potentially attributable to menopause. The current study design does not allow us to examine this possibility, but future qualitative work may be helpful in assessing this.

## Limitations

Although this study provides novel insights into ADHD and the menopause, it is not without limitations which should be acknowledged. Firstly, we have relied on self-reporting ADHD diagnosis and as such it is possible that not all women had received the diagnosis they stated, although there was no benefit to them if they claimed or hid a diagnosis so this would seem unlikely. Secondly, significant demographic differences existed between our three groups, which we attempted to mitigate for statistically but lack of consistent detail regarding other conditions, for example, meant that the extent to which we could do this was limited. Thirdly, our sample lacked ethnic diversity, predominately consisting of White participants. Whilst research shows that Asian, Black, and Hispanic adults have reported receiving less frequent ADHD diagnoses, healthcare visits, and suitable treatment than White adults (Adams et al., 2024) which may indicate a larger pool of White women with ADHD to draw on, ethnic differences have been reported in menopause experiences (Kingsberg et al., 2023) meaning this work may not generalize to more ethnically diverse populations. Fourthly, although most of the scales used showed high reliability, low Cronbach’s alpha values were reported for the sleep and sex subscales of the WHQ. Both subscales have a small number of items which likely explains the low internal consistency, which has been previously reported (Girod et al., 2006). Fifthly, whilst we endeavored to collected detailed information from participants, we were not able to consider some clinical features, for example, we could not differentiate between ADHD presentation types or type of medication, because the majority were on stimulants, meaning a non-stimulant group would have been too small. Similarly, this study focused on women who had not undergone surgical menopause and were not taking MHT or contraceptive hormones. These exclusion criteria were used to ensure as homogenous a sample as possible and one which represented typical menopause without medication confounding effects, but these factors need considering in future research. Finally, although our sample size was adequate for H1 and H2 and for all correlations of the non-ADHD group for H3, some correlations for the ADHD-U and ADHD-M for H3 were likely underpowered and so small or medium effects may have been missed.

In conclusion, this study is, to our knowledge, the first study to explore menopausal experiences in women with ADHD. Whilst the study has not revealed any significant differences in menopausal complaints for women with and without ADHD, it has shown that the relationships between menopausal symptoms and ADHD symptoms may differ in those with and without a diagnosis and those on and off medication. Much more research is needed to understand these relationships, including the effects of medication and it is likely that longitudinal research will be required. It is important to recognize that the overlapping symptoms of ADHD and the menopause may make it difficult to reliably assess either using scale measures. Future research should aim to determine the sensitivity of the different scales in identifying genuine ADHD and menopausal symptoms in women of this age and explore other methods to support valid assessments, such as comprehensive clinical interviews or objective evaluations by clinicians. This has implications for diagnosing ADHD during the menopause transition, although given diagnosis typically does not rely on scales, this is unlikely to be a significant issue. It is more likely to impact research where scales may be utilized more.

## Supplemental Material

sj-docx-1-jad-10.1177_10870547251355006 – Supplemental material for Examining the Link Between ADHD Symptoms and Menopausal Experiences Supplemental material, sj-docx-1-jad-10.1177_10870547251355006 for Examining the Link Between ADHD Symptoms and Menopausal Experiences by Lauren Chapman, Kanak Gupta, Myra S. Hunter and Eleanor J. Dommett in Journal of Attention Disorders
