# Supplementary material for: Examining the Link Between ADHD Symptoms and Menopausal Experiences
Source: J Atten Disord. 2025 Jul 30;29(14):1263–77. doi: 10.1177/10870547251355006 (PMC12569137; doi:10.1177/10870547251355006)
Supplement: sj-docx-1-jad-10.1177_10870547251355006 – Supplemental material for Examining the Link Between ADHD Symptoms and Menopausal Experiences [file sj-docx-1-jad-10.1177_10870547251355006.docx]

Supplementary Table S1: Different conditions recorded by participants. Not all participants provided details of their additional diagnosis and many reported multiple conditions.

| **Category** | **Conditions** |
| --- | --- |
| Neurological and Neurodevelopmental | Autism Spectrum Disorder (ASD)/Asperger’s |
|  | Dyslexia |
|  | Dyspraxia |
|  | Obsessive-Compulsive Disorder (OCD) |
|  | Epilepsy |
|  | Migraine |
|  | Fibromyalgia |
|  | Idiopathic Intracranial Hypertension |
|  | Myopic macular degeneration |
|  | Focal Cemento Osseous Dysplasia |
| Mental Health Conditions | Depression |
|  | Anxiety |
|  | PTSD (Post-Traumatic Stress Disorder) |
|  | Bipolar Disorder |
|  | Borderline Personality Disorder (BPD) |
|  | Emotionally Unstable Personality Disorder |
|  | Avoidant Restrictive Food Intake Disorder (ARFID) |
| Endocrine, Metabolic, and Reproductive | Underactive/overactive Thyroid |
|  | PCOS (Polycystic Ovary Syndrome) |
|  | Diabetes |
|  | Graves' disease |
|  | High cholesterol |
|  | High blood pressure |
|  | Thyroiditis |
|  | Endometriosis |
|  | PMDD (Premenstrual Dysphoric Disorder) |
|  | Adenomyosis |
| Autoimmune and Chronic Inflammatory | Lupus (SLE) |
|  | Rheumatoid arthritis |
|  | MS/ME |
|  | Postural orthostatic tachycardia syndrome (POTS) |
| Gastrointestinal | IBS (Irritable Bowel Syndrome) |
|  | Acid reflux |
|  | Gallstones |
|  | Hiatus Hernia |
| Musculoskeletal | Arthritis |
|  | Ehlers-Danlos Syndrome (EDS) |
|  | Joint Hypermobility Syndrome |
|  | Costochondritis |
|  | Osteoporosis |
|  | Scoliosis |
|  | Degenerative disc disease |
|  | Sciatica |
|  | SpinaBifida |
| Respiratory | Asthma |
|  | Chronic Obstructive Pulmonary Disease (COPD) |
|  | Sleep Apnoea |
|  | Hayfever/ animal fur allergy |
| Oncological | Breast cancer |
| Dermatological | Psoriasis |
|  | Rosacea |
|  | Vitiligo |
|  | Ezcema |
| Miscellaneous | Long Covid |
|  | Raynaud’s |
|  | Gilbert’s syndrome |
|  | Stress oedema |
|  | Meniere's disease |
|  | Congenital defect |
|  | "Sleep issues" |
|  | HIV |

Supplementary Table S2: Main effects of menopause stage on menopausal complaints with significant effects withstanding FDR correction indicated in bold.

| **Menopausal Complaints** | **Subscales** | **Pre**  **M(SD)** | **Peri**  **M (SD)** | **Post**  **M (SD)** | **df** | **F** | ***p*** | **η_p_^2^** | **Post hoc comparisons** |
| --- | --- | --- | --- | --- | --- | --- | --- | --- | --- |
| WHQ | Anxiety/depressed | 2.42 (0.69) | 2.30 (0.67) | 2.33 (0.66) | 2, 632 | 2.590 | .076 | .008 |  |
|  | Wellbeing | 2.75 (0.59) | 2.64 (0.56) | 2.61 (0.59 | 2, 633 | 4,315 | **.014** | .013 | n.s. |
|  | Sleep | 2.22 (0.86) | 2.02 (0.75) | 2.03 (0.79) | 2, 634 | 2.454 | .087 | .008 |  |
|  | Vasomotor | 2.89 (0.93) | 2.49 (0.94) | 2.25 (0.98) | 2, 636 | 19.918 | **<.001** | .059 | Pre > peri (*p* < 0.001); pre > post (*p* < 0.001); peri> post (*p* = 0.019) |
|  | Somatic | 2.30 (0.67) | 2.15 (0.67) | 2.24 (0.67) | 2, 633 | 3.151 | .043 | .010 |  |
|  | Memory/concentration | 1.87 (0.62) | 1.69 (0.57) | 1.80 (0.64) | 2, 633 | 4.483 | **.012** | .014 | Pre > peri (*p* = 0.020) |
|  | Sex | 2.60 (0.73) | 2.43 (0.74) | 2.20 (0.76) | 2, 357 | 2.122 | .121 | .012 |  |
|  | Menstrual | 1.92 (0.73) | 1.97 (0.70) | - | 1, 372 | .079 | .779 | .000 |  |
| MENQoL | Vasomotor | 2.89 (1.94) | 3.56 (2.12) | 3.84 (2.25) | 2, 639 | 9,969 | **<.001** | .030 | Pre < peri (*p* = 0.013); pre < post (*p* < 0.001) |
|  | Psychosocial | 5.09 (1.66) | 5.53 (1.61) | 5.13 (1.70) | 2, 639 | 4.500 | **.011** | .014 | Pre < peri (*p* = 0.047); peri> post (*p* = 0.019) |
|  | Physical | 4.39 (1.53) | 4.75 (1.32) | 4.77 (1.38) | 2, 639 | 2.535 | .080 | .008 |  |
|  | Sexual | 3.28 (2.16) | 3.51 (2.07) | 3.95 (2.23) | 2, 639 | 1.097 | .335 | .003 |  |
| HFRS | HF frequency/week | 5.17 (6.13) | 12.51 (16.12) | 17.69 (23.89) | 2, 309 | 5.387 | **.005** | .034 | Pre< post (*p* < 0.001) |
|  | NS frequency/week | 4.73 (4.73) | 8.91 (10.57) | 11.38 (11.04) | 2, 315 | 5.435 | **.005** | .033 | Pre< post (*p* < 0.001) |
|  | HF Severity | 1.32 (1.25) | 1.05 (1.08) | 0.90 (0.82) | 2, 371 | 1.920 | .148 | .010 |  |
|  | NS Severity | 0.93 (0.93) | 1.0 (0.90) | 1.13 (0.84) | 2, 363 | .040 | .342 | .005 |  |
|  | Problem Rating | 3.22 (1.99) | 3.79 (2.25) | 4.15 (2.49) | 2, 370 | 3.570 | .029 | .019 | Pre< post (*p* = 0.044) |
| HFDIS | Total | 2.53 (2.40) | 3.33 (2.71) | 3,91 (2.89) | 2, 350 | 4.262 | **.015** | .009 | Pre< post (*p* = 0.007) |

Supplementary Table S3: Interaction effects for ADHD diagnosis (ADHD vs non-ADHD) and menopause status. Note that no interactions were significant after application of FDR correction.

| **Menopausal Complaints** | **Subscales** | **df** | **F** | ***p*** | η_p_^2^ |
| --- | --- | --- | --- | --- | --- |
| WHQ | Anxiety/depressed | 2, 632 | .835 | .434 | .003 |
|  | Wellbeing | 2, 633 | 3.186 | .042 | .010 |
|  | Sleep | 2, 634 | 2.483 | .084 | .008 |
|  | Vasomotor | 2, 636 | .020 | .980 | .000 |
|  | Somatic | 2, 633 | 1.129 | .324 | .004 |
|  | Memory/concentration | 2, 633 | .515 | .597 | .002 |
|  | Sex | 2, 357 | .738 | .479 | .004 |
|  | Menstrual | 2, 372 | .280 | .597 | .001 |
| MENQoL | Vasomotor | 2, 639 | .068 | .934 | .000 |
|  | Psychosocial | 2, 639 | .562 | .570 | .002 |
|  | Physical | 2, 639 | .267 | .766 | .001 |
|  | Sexual | 2, 639 | 1.174 | .310 | .004 |
| HFRS | HF frequency/week | 2, 309 | .512 | .600 | .003 |
|  | NS frequency/week | 2, 315 | 2.110 | .122 | .013 |
|  | HF Severity | 2, 371 | .203 | .817 | .001 |
|  | NS Severity | 2, 363 | 1.009 | .365 | .006 |
|  | Problem Rating | 2, 370 | 1.898 | .151 | .010 |
| HFDIS | Total | 2, 350 | 1.545 | .215 | .009 |

Supplementary Table S4: Interaction effects for ADHD status (ADHD-medicated, ADHD-unmedicated, non-ADHD) and menopause status. Note that no interactions remained significant after application of FDR correction.

| **Menopausal Complaints** | **Subscales** | **df** | **F** | ***p*** | η_p_^2^ |
| --- | --- | --- | --- | --- | --- |
| WHQ | Anxiety/depressed | 4, 629 | 2.004 | .092 | .013 |
|  | Wellbeing | 4, 630 | 3.045 | .017 | .019 |
|  | Sleep | 4, 631 | 1.264 | .283 | .008 |
|  | Vasomotor | 4, 633 | .198 | .940 | .001 |
|  | Somatic | 4, 630 | 1.558 | .184 | .010 |
|  | Memory/concentration | 4, 630 | 1.004 | .405 | .006 |
|  | Sex | 4, 354 | .445 | .776 | .005 |
|  | Menstrual | 2, 370 | 1.095 | .336 | .006 |
| MENQoL | Vasomotor | 4, 638 | .430 | .787 | .003 |
|  | Psychosocial | 4, 638 | 2.536 | .039 | .016 |
|  | Physical | 4, 638 | .246 | .912 | .002 |
|  | Sexual | 4, 638 | 1.031 | .390 | .006 |
| HFRS | HF frequency/week | 4, 308 | 1.009 | .403 | .013 |
|  | NS frequency/week | 4, 314 | 1.249 | .290 | .016 |
|  | HF Severity | 2, 306 | 2.078 | .084 | .026 |
|  | NS Severity | 2, 306 | 1.596 | .175 | .020 |
|  | Problem Rating | 4, 369 | 1.118 | .348 | .012 |
| HFDIS | Total | 4, 349 | 1.191 | .314 | .013 |

Supplementary Table S5: Comparisons of correlations between the three ADHD groups for the correlations of ASRS score with menopausal complaints. P-values shown in bold indicate that the group differences remained statistically significant after FDR correction.

| **Menopausal Complaints** | **Subscales** | **Non-ADHD/ADHD-M** | | **Non-ADHD/ADHD-U** | | **ADHD-M/ADHD-U** | |
| --- | --- | --- | --- | --- | --- | --- | --- |
|  |  | **z** | ***p*** | **z** | ***p*** | **z** | ***p*** |
| WHQ | Anxiety/depressed | -2.414 | **.008** | -1.27 | .102 | -1.068 | .143 |
|  | Wellbeing | 0.236 | .407 | 1.813 | .190 | 1.178 | .119 |
|  | Sleep | -0.16 | .437 | 0.333 | .370 | 0.452 | .326 |
|  | Vasomotor | 0.164 | .435 | -1.11 | .134 | 1.178 | .119 |
|  | Somatic | 1.946 | .026 | 1.773 | .038 | -0.29 | .386 |
|  | Memory/concentration | 2.478 | **.007** | 2.458 | **.007** | -0.211 | .416 |
|  | Sex | 1.236 | .108 | 1.753 | .040 | 0.217 | .414 |
|  | Menstrual | 0.014 | .494 | 0.566 | .286 | 0.424 | .336 |
| MENQoL | Vasomotor | 0.196 | .422 | 0.879 | .190 | 0.504 | .307 |
|  | Psychosocial | 2.189 | .014 | 3.232 | **.001** | 0.617 | .269 |
|  | Physical | 1.059 | .145 | 2.125 | .017 | 0.725 | .234 |
|  | Sexual | 0.561 | .287 | 0.763 | .223 | 0.108 | .457 |
| HFRS | HF frequency/week | 0.88 | .189 | -0.27 | .393 | -0.941 | .173 |
|  | NS frequency/week | 0.162 | .436 | -1.879 | .030 | -1.256 | .105 |
|  | HF Severity | -0.077 | .469 | 1.616 | .053 | 1.297 | .097 |
|  | NS Severity | -0.385 | .35 | -0.822 | .205 | -0.309 | .378 |
|  | Problem Rating | 0.21 | .417 | 0.549 | .292 | 0.244 | .404 |
| HFDIS | Total | -0.246 | .403 | 2.139 | .016 | 1.824 | .034 |
